# Outcomes and predictors of patients with moderate or severe functional mitral regurgitation and nonischemic dilated cardiomyopathy

**DOI:** 10.1002/clc.24067

**Published:** 2023-06-15

**Authors:** Jiangtao Li, Xiang Wei

**Affiliations:** ^1^ Division of Cardiothoracic and Vascular Surgery, Tongji Hospital, Tongji Medical College Huazhong University of Science and Technology Wuhan Hubei China; ^2^ Key Laboratory of Organ Transplantation Ministry of Education Wuhan Hubei China; ^3^ NHC Key Laboratory of Organ Transplantation Ministry of Health Wuhan Hubei China

**Keywords:** functional mitral regurgitation, medical therapy, mitral valve repair, nonischemic dilated cardiomyopathy, prognosis, risk factors

## Abstract

**Background:**

Patients with functional mitral regurgitation (FMR) and nonischemic dilated cardiomyopathy (DCM) are associated with high mortality.

**Objectives:**

Our study aimed to compare the clinical outcomes between different treatment strategies and identify predictors associated with the adverse outcomes.

**Methods:**

A total of 112 patients with moderate or severe FMR and nonischaemic DCM were included in our study. The primary composite outcome was all‐cause death or unplanned hospitalization for heart failure. The secondary outcomes were individual components of the primary outcome and the cardiovascular death.

**Results:**

In this study, the primary composite outcome occurred in 26 patients (44.8%) in mitral valve repair (MVr) group and 37 patients (68.5%) in medical group (hazard ratio [HR], 0.28; 95% confidence interval [CI], 0.14–0.55; *p* < .001). The 1‐, 3‐, and 5‐year survival rates for patients with MVr were 96.6%, 91.8%, and 77.4%, respectively, which were significantly higher than that of medical group: 81.2%, 71.9%, and 65.1%, respectively (HR, 0.32; 95% CI, 0.12–0.87; *p* = .03). Left ventricular ejection fraction (LVEF) < 41.5% (*p* < .001) and atrial fibrillation (*p* = .02) were independently associated with the primary outcome. LVEF < 41.5% (*p* = .007), renal insufficiency (*p* = .003), and left ventricular end‐diastolic diameter > 66.5 mm (*p* < .001) were independently associated with heightened risk for all‐cause death.

**Conclusion:**

Compared with medical therapy, MVr was associated with a better prognosis in patients with moderate or severe FMR and nonischemic DCM. We observed that LVEF < 41.5% was the only independent predictor of the primary outcome and all individual components of secondary outcomes.

## Graphical abstract

Outcomes and predictors of patients with moderate or severe functional mitral regurgitation and nonischemic dilated cardiomyopathy. CI, confidence interval; HR, hazard ratio; LVEF, left ventricular ejection fraction; MVr, mitral valve repair.

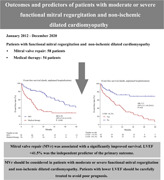

**Abbreviations**
DCM: dilated cardiomyopathy
FMR: functional mitral regurgitation
LVEF: left ventricular ejection fraction
MR: mitral regurgitation
MV: mitral valve
MVr: mitral valve repair

## 1 INTRODUCTION

Functional mitral regurgitation (MR), which occurs in the setting of left ventricular remodeling and structurally normal mitral valve (MV), is frequently occurred in patients with ischemic cardiomyopathy and nonischemic dilated cardiomyopathy (DCM). Despite medical and surgical therapeutic advances,^1^ functional mitral regurgitation (FMR) is associated with high morbidity and mortality.^2^, ^3^ For the patients with moderate or severe FMR, the prognosis is grave with the rates of all‐cause death ranging from 15% to 30% in 1 year.^4^

With controversial outcomes, it remains unclear whether mitral valve repair (MVr) could improve the prognosis in patients with FMR. Additionally, for patients with FMR and nonischemic DCM, the current European Society of Cardiology/European Association for Cardio‐Thoracic Surgery and American College of Cardiology/American Heart Association guidelines consider MVr as a class IIb recommendation.^2^, ^5^

FMR is frequently observed in patients with nonischemic DCM and promotes further the process of left ventricular remodeling (MR begets MR). Therefore, it is logical to hypothesize that the correction of MR may improve the prognosis among patients with nonischemic DCM. However, current studies reporting the survival after mitral regurgitation repair for FMR are controversial.^6^, ^7^, ^8^, ^9^ Several studies have reported that patients with MVr had better survival.^6^, ^7^ For example, Bolling et al.^10^ reported that MVr improved the prognosis of patients with severe FMR, the actuarial survivals of whom were 82% and 72% at 1 and 2 years. However, others suggested that there were no survival advantages of MVr.^8^, ^9^ Most published data on this procedure analyzed the outcomes in patients with ischemic cardiomyopathy or combined populations of ischemic cardiomyopathy and nonischemic DCM.^6^, ^8^, ^9^, ^11^, ^12^, ^13^ Moreover, because of the different pathophysiology and the bias of an additional surgical procedure (with/without revascularization), we hypothesized that the MVr for patients with FMR and nonischemic DCM could somewhat be different from those with ischemic MR.

The aims of our study were as follows: (i) describe our practices in the management of patients with moderate or severe FMR and nonischemic DCM at our academic center; (ii) compare the clinical outcomes between different treatment strategies; (iii) identify risk factors associated with the adverse outcomes.

## 2 MATERIALS AND METHODS

### 2.1 Patients selection and definitions

Our study complied with the 2013 Declaration of Helsinki. This study was approved by the local review board (No. 2021‐S239) and the written informed consent was waived. Between January 2012 and December 2020, patients with moderate or severe FMR and nonischaemic DCM were retrospectively identified after the echocardiographic and clinical evaluation from our institution database.

Mitral valve morphologic description of abnormal, thickening, myxoma, or prolapse‐defined organic MR. The severity of MR was divided into none, mild, moderate, and severe.^14^ In the absence of organic MV apparatus disease, MR was defined as functional. Inclusion criteria were patients ≥18 years of age with MR and DCM. Exclusion criteria for our study were as follows: (1) patients with organic mitral valve disease; (2) patients with aortic regurgitation or stenosis; (3) patients with previous myocardial infarction, previous coronary artery bypass grafting or percutaneous intervention, or coronary artery disease, which was defined as ≥50% stenosis of a coronary vessel; (4) patients with prior history of MV surgery or cardiac transplant; (5) patients with MR less than moderate; (6) patients lost to follow‐up. Therefore, a total of 112 patients (58 with MVr and 54 with medical therapy) were included for final analysis in this study.

### 2.2 Treatment

It was up to the Heart Team (including clinical cardiologist, cardiac surgeon, and imaging specialist with expertise in cardiovascular imaging) to decide whether to perform MVr based on the patient's characteristics, anatomical suitability, and the willingness of patients. Patients who refused or decided not to undergo surgery were classified as the medical group. Both surgical and medical patients were treated with standard guideline‐directed medications,^15^ including angiotensin‐converting enzyme inhibitors (ACEIs), angiotensin receptor blockers (ARBs), beta‐blockers, aldosterone antagonists, and diuretics.

All operations were performed with a standard cardiopulmonary bypass of mild hypothermia. The exposure of MR was achieved by either the left atrial (Waterston's groove) or biatrial approach. MVr was accomplished using restrictive mitral ring annuloplasty.^16^ The MV annulus was measured and the ring was implanted, downsizing the ring by two sizes.^17^

### 2.3 Follow‐up and outcomes

The follow‐up data were verified through inpatient and outpatient medical records, telephone contact with the patients, or interviews with relatives. The follow‐up ended in September 2021, or when the patient died. The primary composite outcome of our study was all‐cause death or unplanned hospitalization for heart failure (such as admissions due to decompensation, implanting the biventricular pacemaker, receiving MVr, or cardiac transplant). The secondary outcomes were individual components of the primary outcome and the cardiovascular death.

### 2.4 Statistical analysis

The Cox proportional hazards regression model was built to demonstrate that the association between treatment strategies and survival was independent of differences in baseline demographic and clinical variables. The adjustment variables included age, sex, heart rates, systolic blood pressure, New York Heart Association Class III/IV, hypertension, dyslipidemia, diabetes mellitus, renal insufficiency, stroke, history of prior non‐MVr, left bundle branch block, atrial fibrillation, serum creatinine level, hemoglobin, left atrial diameter, left ventricular end‐diastolic diameter (LVEDD), left ventricular ejection fraction (LVEF), MR severity and tricuspid regurgitation (≥moderate). The event‐free survival curves were analyzed by the Kaplan–Meier method and the differences in survival estimates were tested by the logarithmic ranking test. Cox's hazard regression model was built to identify predictors of the primary outcome and secondary outcomes. The results of the Cox proportional hazards analysis were presented as hazard ratio (HR), 95% confidence interval (CI), and *p* value.

Patients in the medical group were defined as the reference group in our analyses. Differences were deemed to be statistically significant with a two‐sided *p* < .05. All statistical analyses were performed using SPSS version 26.

## 3 RESULTS

### 3.1 Baseline characteristics

**Figure 1:**
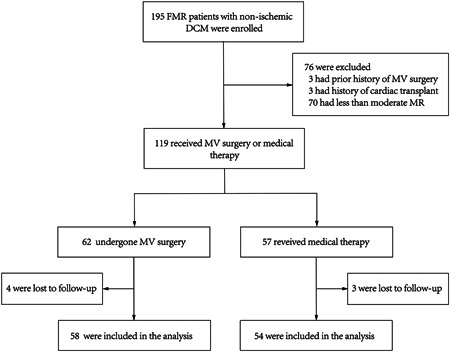
Flow chart of patients with moderate or severe FMR and nonischemic DCM. DCM, dilated cardiomyopathy; FMR, functional mitral regurgitation; MR, mitral regurgitation; MVr, mitral valve surgery.

In total, 112 patients (55.4 ± 12.5 years old, 46.4% male) with moderate or severe FMR and nonischemic DCM were evaluated in our study. The median follow‐up was 38 months (interquartile range [IQR], 21–68 months). Among those 112 patients, MVr was pursued in 58 patients, and 54 patients received medical therapy (Figure 1).

The baseline and operative characteristics of patients divided according to the treatment strategies are shown in Supporting Information: Table S1. Though not significantly, patients in the medical group presented more frequently with male sex, tricuspid regurgitation (≥moderate), and higher serum creatinine level compared with the MVr group.

In the MVr group, 21 patients (36.2%) underwent concomitant surgical tricuspid valve annuloplasty, and the atrial maze procedure was performed in 10 patients (17.2%) for atrial fibrillation. The mean cardiopulmonary bypass and cross‐clamping times were 117.1 ± 38.0 and 64.7 ± 26.2 min, respectively.

### 3.2 Survival analysis

**Figure 2:**
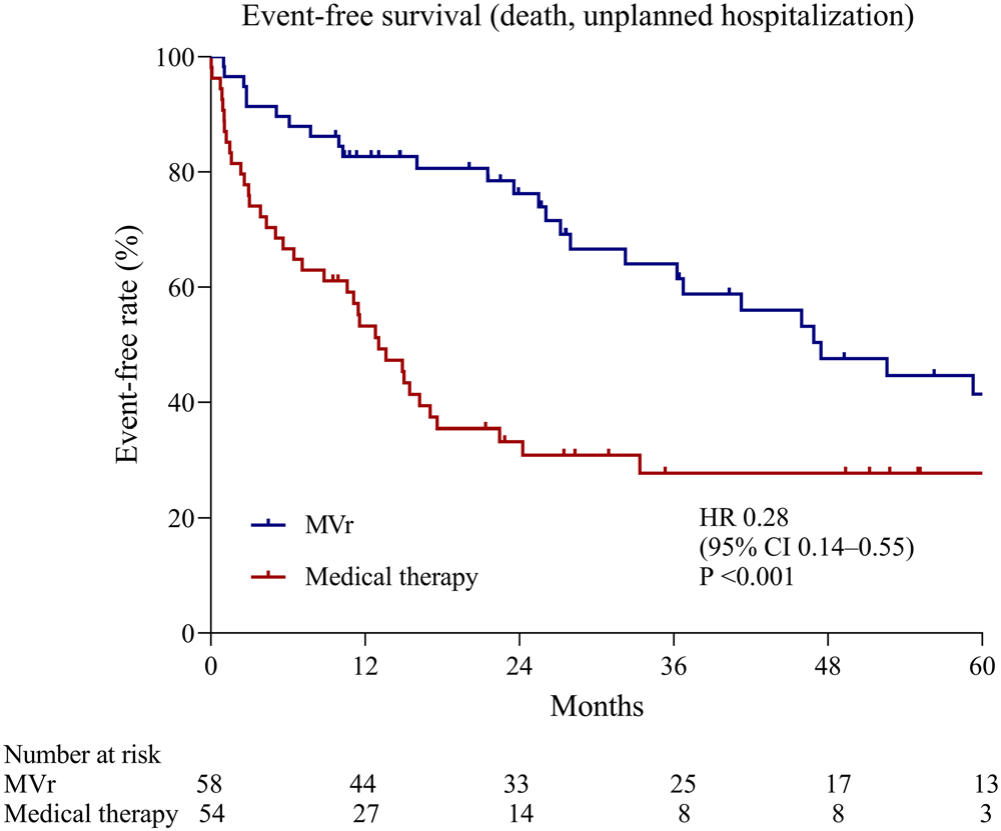
Kaplan–Meier estimates of survival without the primary outcome event. Shown are estimates of the probability of survival without the primary outcome event (all‐cause cause or unplanned hospitalization for heart failure) in the mitral valve repair group and the medical group. CI, confidence interval; HR, hazard ratio.

In this study, the in‐hospital mortality was 1.7% (one patient) in the MVr group and 1.9% (one patient) in the medical group. The primary composite outcome of death from any cause or unplanned hospitalization for heart failure occurred in 26 patients (44.8%) in the MVr group and 37 patients (68.5%) in the medical group (HR, 0.28; 95% CI, 0.14–0.55; *p* < .001) (Supporting Information: Table S2). As shown in Figure 2, compared with medical therapy, the treatment of MVr was associated with significantly better event‐free survival from the primary outcome with the 3‐year event‐free survival rate of 64.1% in patients with MVr and 27.8% in medically treated patients.

**Figure 3:**
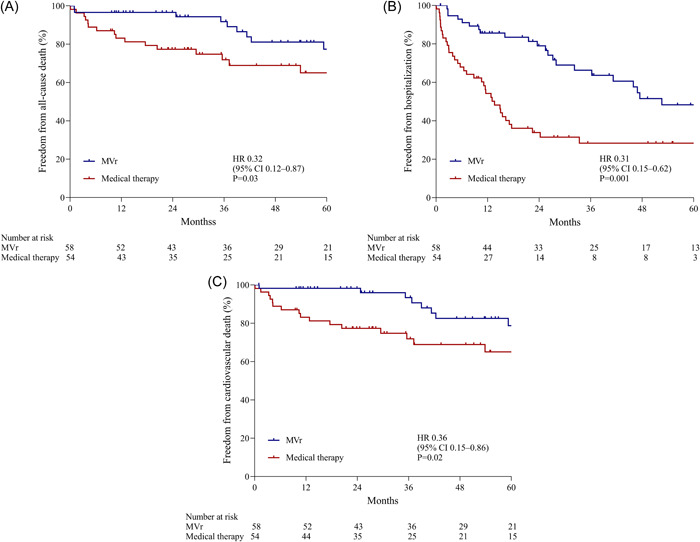
Kaplan–Meier estimates for the individual component of the primary outcome and cardiovascular death. (A) All‐cause death: the probability of survival (considering death from any cause) in the mitral valve repair group and medical group. (B) Hospitalization for heart failure: the probability of freedom from unplanned hospitalization for heart failure in the mitral valve repair group and medical group. (C) Cardiovascular death: the probability of survival (considering cardiovascular death) in the mitral valve repair group and the medical group. CI, confidence interval; HR, hazard ratio.

During the follow‐up, a total of 10 (17.2%) deaths occurred in the MVr group, as compared with 20 (37.0%) in the medical group. The 1‐, 3‐, and 5‐year survival rates for patients with MVr were 96.6%, 91.8%, and 77.4%, respectively, which were significantly higher than the survival of the medical group: 81.2%, 71.9%, and 65.1%, respectively (HR, 0.32; 95% CI, 0.12–0.87; *p* = .03) (Figure 3A). Twenty‐two patients (37.9%) who received the MVr had at least one unplanned hospitalization for heart failure, as compared with 36 patients (66.7%) in the medical therapy group (HR, 0.31; 95% CI, 0.15–0.62; *p* < .001) (Figure 3B). Event‐free survival from cardiovascular death was significantly higher in the MVr group than that in the medical group (HR, 0.36; 95% CI, 0.15–0.86; *p* = .02) (Figure 3C).

### 3.3 Predictors for the primary outcome and secondary outcomes

**Figure 4:**
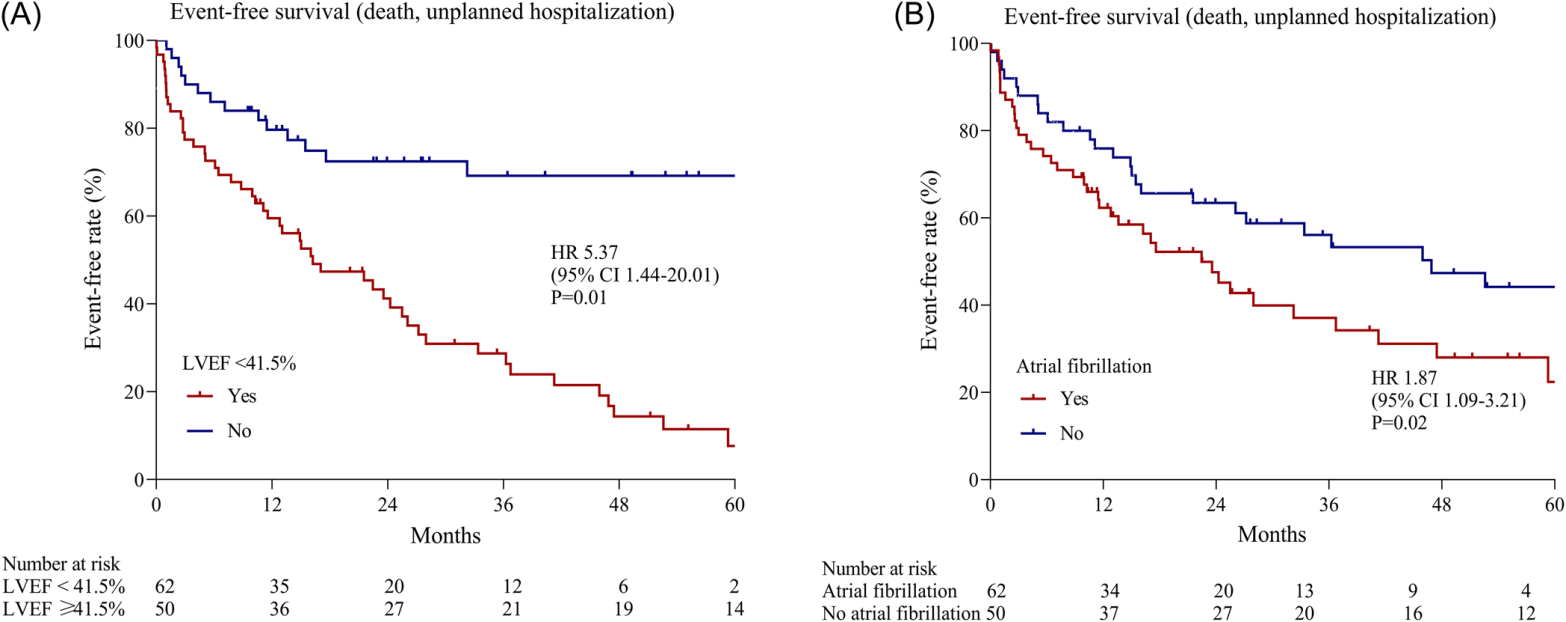
Kaplan–Meier estimates for the primary outcome according to LVEF and atrial fibrillation. Significantly lower event‐free survival rates were observed in patients with LVEF < 41.5% (A) and atrial fibrillation (B). CI, confidence interval; HR, hazard ratio; LVEF, left ventricular ejection fraction.

The Cox regression model for predicting the primary outcome and secondary outcomes in MVr patients was shown in Supporting Information: Table S3. Multivariate analysis showed that LVEF < 41.5% (HR, 3.71; 95% CI, 2.02–6.82; *p* < .001) and atrial fibrillation (HR, 1.87; 95% CI, 1.09–3.21; *p* = .02) were independently associated with the primary outcome (Figure 4).

**Figure 5:**
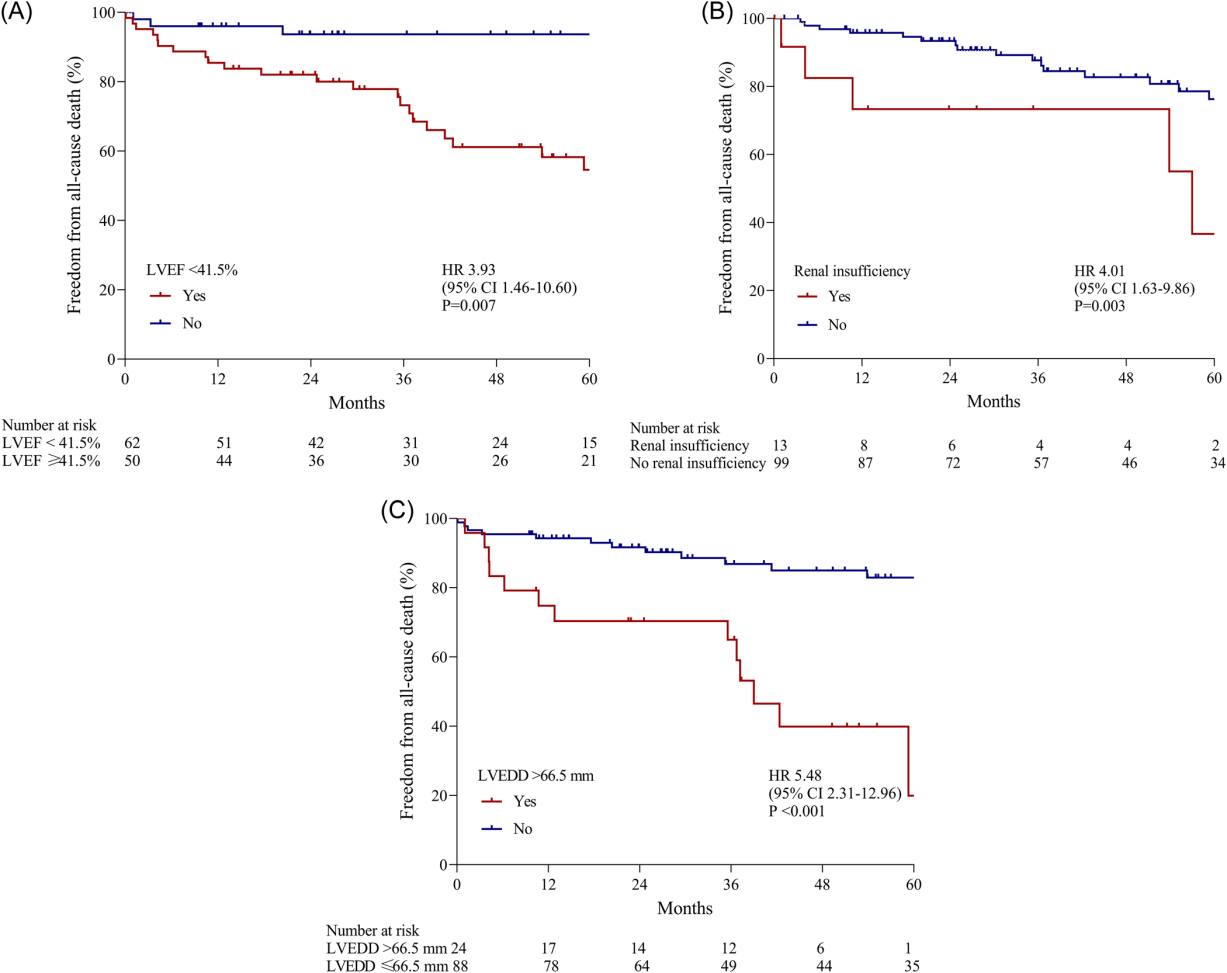
Kaplan–Meier estimates for all‐cause death according to LVEF, renal insufficiency, and LVEDD. Significantly lower survival rates were observed in patients with LVEF < 41.5% (A), renal insufficiency (B), and LVEDD > 66.5 mm (C). CI, confidence interval; HR, hazard ratio; LVEDD, left ventricular end‐diastolic diameter; LVEF, left ventricular ejection fraction.

LVEF < 41.5% (HR, 3.93; 95% CI, 1.46–10.60; *p* = .007), renal insufficiency (HR, 4.01; 95% CI, 1.63–9.86; *p* = .003), and LVEDD > 66.5 mm (HR, 5.48; 95% CI, 2.31–12.96; *p* < .001) were independent predictors of all‐cause death. Significantly lower survival rates were observed in patients with LVEF < 41.5%, renal insufficiency, and LVEDD > 66.5 mm (Figure 5).

LVEF < 41.5% (HR, 3.86; 95% CI, 2.06–7.22; *p* < .001) was the only independent predictor of unplanned hospitalization for heart failure. Significantly lower survival rates were observed in patients with LVEF < 41.5% (Supporting Information: Figure S1).

LVEF < 41.5% (HR, 6.02; 95% CI, 1.93–18.78; *p* = .002) and LVEDD > 66.5 mm (HR, 2.99; 95% CI, 1.25–7.16; *p* = .01) were independent predictors of cardiovascular death. Significantly lower survival rates were observed in patients with LVEF < 41.5% and LVEDD > 66.5 mm (Supporting Information: Figure S2).

## 4 DISCUSSION

In this study, we compared the outcomes between MVr group and the medical group and investigated the predictors of adverse outcomes in patients with moderate or severe FMR and nonischemic DCM. Our results showed that the MVr patients had better event‐free survival than those with medical therapy. We observed that LVEF < 41.5% was the only independent predictor of the primary outcome (all‐cause death or unplanned hospitalization for heart failure) and all individual components of secondary outcomes (all‐cause death, unplanned hospitalization for heart failure, or cardiovascular death).

FMR is a frequent presentation in patients with nonischemic DCM, as well as those with ischemic cardiomyopathy. It was reported that patients with FMR and nonischemic DCM are associated with multiple comorbidities and poor long‐term survival, with only 30% of the 5‐year survival.^2^, ^4^, ^18^, ^19^ With the majority of studies focusing on the prognostic impact of ischemic MR or mixed FMR including ischemic cardiomyopathy and nonischemic DCM,^7^, ^20^, ^21^, ^22^ there are scarce reports demonstrating the outcomes in patients with FMR and nonischemic DCM. Additionally, it remains controversial whether MVr improves survival.

The best strategy to treat FMR in nonischemic DCM is widely debated. MVr has been reported to be a good solution for patients with FMR and nonischemic DCM.^23^ It was noticed that short‐term outcomes were encouraging with the relief of heart failure and stable MV function in some patients, whereas long‐term survival has not been improved. Moreover, it has been reported that there was no significant survival benefit conferred by MVr for patients with FMR and nonischemic DCM.^8^ However, Chung et al.^18^ reported that the correction of MR could improve the prognosis in these patients. In addition, Stone et al.^24^ in a cardiovascular outcomes assessment of the MitraClip percutaneous therapy (COPAT) study reported that transcatheter mitral‐valve repair using the MitraClip device resulted in a lower rate of heart failure‐related hospitalization and lower all‐cause death within 2 years of follow‐up than medical therapy alone. In our study, MVr was associated with a lower rate of the composite outcome of death from any cause or unplanned hospitalization for heart failure than medical therapy. Besides, compared with the medical group, the rates of all‐cause death, unplanned hospitalization for heart failure, and cardiovascular death were significantly lower in the MVr group.

With the hospital mortality of 1.7% in patients who received MVr, our hospital mortality was comparable with the results in the previous studies.^24^, ^25^, ^26^ However, in our study, the 1‐, 3‐, and 5‐year survival rates for patients with MVr were 96.6%, 91.8%, and 77.4%, respectively, which were significantly higher than those reports.^24^, ^25^, ^26^ It was noticed that the MVr patients in this study had baseline higher LVEF and smaller LVEDD than those in the previously reported literature.^8^, ^19^, ^24^, ^25^, ^26^, ^27^ Further, the MR severity of our patients was less than those undergoing transcatheter mitral‐valve repair.^24^ Given the high rate of mortality in previous studies, MVr might have been performed too late in the course of the progression of heart failure. Therefore, the potential benefit of MVr might be diminished in those with more serious illnesses, such as patients with lower LVEF and larger LVEDD. On the contrary, patients in our study might receive MVr in the early stage of the nonischemic DCM. The better prognosis of the MVr may suggest that the progression of heart failure slowed down by the surgical correction of MR. Our findings are encouraging in terms of the role of MVr in patients with moderate or severe FMR and nonischemic DCM but need to be validated in larger patient populations and randomized controlled trials.

To achieve a better outcome for nonischemic FMR patients undergoing MVr, it might be useful to identify the predictors for the primary outcome and secondary outcomes. As expected, a low LVEF was a powerful predictor of adverse outcomes. LVEF is the most commonly utilized parameter of cardiac function and patients with higher LVEF were associated with a linear decrease in mortality.^28^ Our findings regarding LVEF were consistent with the result from other studies, in which LVEF was a strong predictor of adverse outcomes.^4^, ^29^, ^30^, ^31^

It has been reported that impaired renal function was an independent predictor of all‐cause death in patients with heart failure.^32^ According to our results, renal insufficiency (defined as estimated glomerular filtration rate < 60 mL/min/1.73 m^2^) was independently associated with heightened risk for all‐cause death. As in previous studies, reduced kidney function seems to associate with worse outcomes in patients with heart failure.^31^, ^32^

Apart from LVEF and renal insufficiency, some researchers have reported that the poor survival for patients with FMR and nonischemic DCM was related to MR severity, New York Heart Association class, and age.^19^, ^29^, ^33^ In the present study, MR severity and New York Heart Association class were not the predictors for adverse outcomes in our study. It was observed that patients with atrial fibrillation were associated with a higher rate of the composite outcome of all‐cause death or unplanned hospitalization for heart failure. Besides, LVEDD > 66.5 mm was found to be a significant independent predictor of all‐cause death and cardiovascular death.

### 4.1 Limitations

First, the observational data could only represent our single‐center experience and are thus influenced by inherent biases. Second, there might be potential bias in the treatment strategies, which serves as an important limitation. Therefore, we attempted to adjust for differences by using a multivariate Cox regression model to minimize treatment bias. It is important to note, however, that this method was not a complete substitute for a true randomized comparison and some unrecognized confounding factors might have affected the results. Finally, the number of patients in this study is relatively small, which may be insufficient to recognize the potential predictors for adverse outcomes. Additionally, those predictors with moderate relevant risk might have been ignored. Our findings, therefore, require further confirmation in a larger size of the data set and randomized trials.

## 5 CONCLUSION

MVr in contrast to medical therapy was associated with a better prognosis in patients with moderate or severe FMR and nonischemic DCM. We observed that LVEF < 41.5% was the only independent predictor of the primary outcome (all‐cause death or unplanned hospitalization for heart failure) and all individual components of secondary outcomes (all‐cause death, unplanned hospitalization for heart failure, or cardiovascular death).

## CONFLICT OF INTEREST STATEMENT

The authors declare no conflict of interest.

## Supporting information

Supporting information.

Supporting information.

## Data Availability

The data that support the findings of this study are available on request from the corresponding author. The data are not publicly available due to privacy or ethical restrictions.
