# Supplementary material for: Outcomes and predictors of patients with moderate or severe functional mitral regurgitation and nonischemic dilated cardiomyopathy
Source: Clin Cardiol. 2023 Jun 15;46(8):922–9. doi: 10.1002/clc.24067 (PMC10436791; doi:10.1002/clc.24067)
Supplement: Supplementary file 1 — Supporting information. [file CLC-46-922-s002.docx]

**Supplemental Figures**

**Figure S1.** Kaplan-Meier estimates for unplanned hospitalization for heart failure according to LVEF


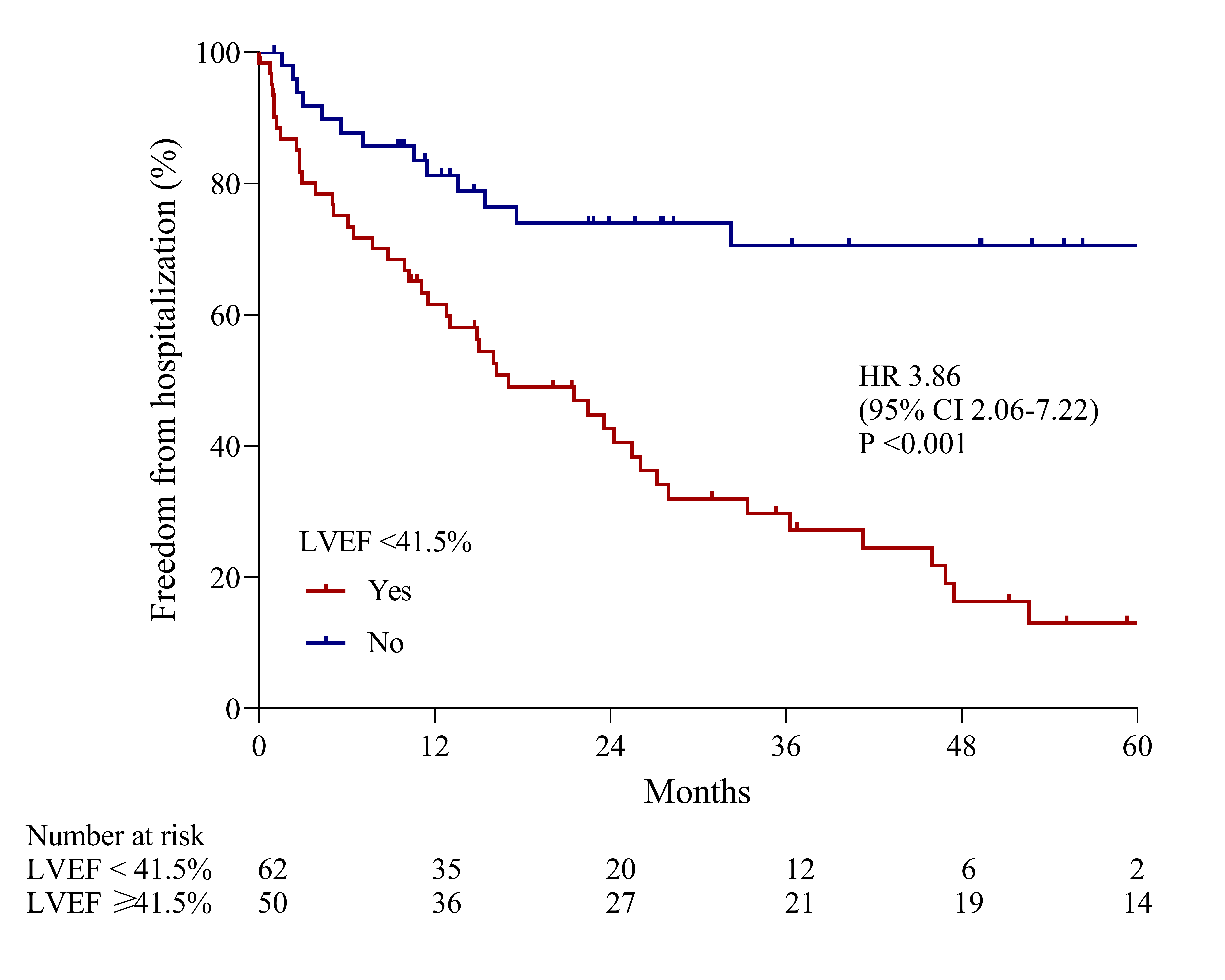


Significantly lower event-free survival rates were observed in patients with LVEF <41.5%. HR, hazard ratio; CI, confidence interval; LVEF, left ventricular ejection fraction.

**Figure S2.** Kaplan-Meier estimates for cardiovascular death according to LVEF and LVEDD




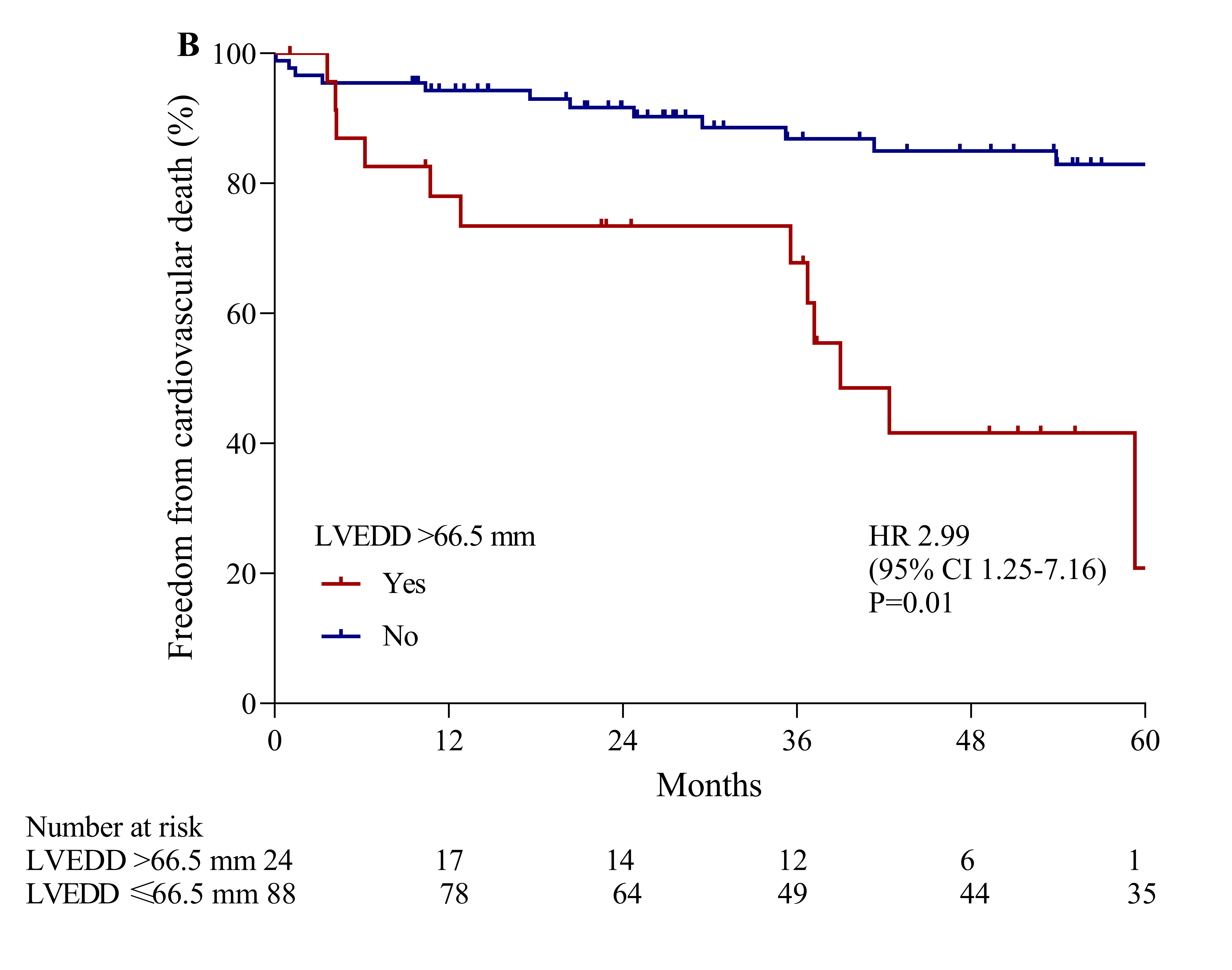


Significantly lower event-free survival (considering cardiovascular death) rates were observed in patients with LVEF <41.5% (A) and LVEDD >66.5 mm (B). HR, hazard ratio; CI, confidence interval; LVEF, left ventricular ejection fraction; LVEDD, left ventricular end-diastolic diameter.
