# Supplementary material for: Outcomes and predictors of patients with moderate or severe functional mitral regurgitation and nonischemic dilated cardiomyopathy
Source: Clin Cardiol. 2023 Jun 15;46(8):922–9. doi: 10.1002/clc.24067 (PMC10436791; doi:10.1002/clc.24067)
Supplement: Supplementary file 2 — Supporting information. [file CLC-46-922-s001.docx]

**Supplemental Tables**

Table S1. Baseline and operative characteristics of the patients.†

| Variables | MVr group (n=58) | Medical group (n=54) | P-value |
| --- | --- | --- | --- |
| Age, year | 54.0 ± 12.6 | 56.8 ± 12.3 | 0.24 |
| Male sex | 23 (39.7) | 29 (53.7) | 0.14 |
| Dyspnea | 52 (89.7) | 68(88.9) | 0.90 |
| NYHA III/IV‡ | 46 (79.3) | 40 (74.1) | 0.51 |
| Heart rate, beats/min | 84.3 ± 13.6 | 85.1 ± 15.4 | 0.76 |
| Systolic blood pressure, mmHg | 123.3 ± 16.9 | 124.5 ± 19.4 | 0.73 |
| Medical and surgical history | | | |
| Hypertension | 25 (43.1) | 26 (48.1) | 0.59 |
| Dyslipidemia | 20(34.5) | 18 (33.3) | 0.90 |
| Diabetes | 8 (13.8) | 7 (13.0) | 0.90 |
| Renal insufficiency | 6 (10.3) | 7 (13.0) | 0.67 |
| Stroke | 5 (8.6) | 1 (1.9) | 0.24 |
| Prior non-mitral valve repair | 6 (10.3) | 5 (9.3) | 0.85 |
| Atrial fibrillation | 33 (56.9) | 29 (53.7) | 0.73 |
| Left bundle branch block | 2 (3.4) | 2 (3.7) | >0.99 |
| Serum creatinine level (μmol/L) | 79.1 ± 36.1 | 88.4 ± 39.8 | 0.20 |
| Hemoglobin, g/L | 127.7 ± 20.0 | 125.3 ± 25.4 | 0.58 |
| Beta-blockers | 32 (55.2) | 29 (55.8) | 0.88 |
| ACEI/ARBs | 34 (58.6) | 32 (61.5) | 0.95 |
| Diuretics | 45 (77.6) | 43 (82.7) | 0.79 |
| Aldosterone antagonists | 46 (79.3) | 42 (80.8) | 0.84 |
| Echocardiographic characteristics | | | |
| Left atrial diameter, mm | 51.0 ± 12.1 | 49.7 ± 11.2 | 0.56 |
| LVEDD, mm | 60.5 ± 7.3 | 60.9 ± 7.0 | 0.77 |
| Left ventricular ejection fraction, % | 41.0 ± 9.2 | 39.4 ± 10.3 | 0.40 |
| Mitral regurgitation severity |  |  | 0.78 |
| Moderate | 22 (37.9) | 20 (37.0) |  |
| Severe | 36 (62.1) | 34 (63.0) |  |
| Tricuspid regurgitation (≥ moderate) | 24 (41.4) | 14 (25.9) | 0.08 |
| Concomitant procedure | | | |
| Tricuspid valve annuloplasty | 28 (36.2) | NA |  |
| Atrial maze | 10 (17.2) | NA |  |
| Duration of aortic cross-clamping, min | 64.7 ± 26.2 | NA |  |
| Duration of cardiopulmonary bypass, min | 117.1 ± 38.0 | NA |  |

† Plus-minus values are means ± standard deviation. Categorical variables are expressed as numbers (%). ACEI, angiotensin-converting enzyme inhibitor; ARB, angiotensin receptor blocker; LVEDD, left ventricular end-diastolic diameter; NA, not applicable; MVr, mitral valve repair.

‡ New York Heart Association (NYHA) classes range from I to IV, with higher classes indicating worse conditions.

Table S2. Primary outcome and secondary outcomes at follow-up.

| Outcome | MVr group  (n=58) | Medical group  (n=54) | Hazard Ratio  (95% CI)* | P-value |
| --- | --- | --- | --- | --- |
| Composite primary outcome: death from any cause or unplanned hospitalization for heart failure | 26 (44.8) | 37 (68.5) | 0.28 (0.14-0.55) | < 0.001 |
| Secondary outcomes† |  |  |  |  |
| Death from any cause | 10 (17.2) | 20 (37.0) | 0.32 (0.12-0.87) | 0.03 |
| Unplanned hospitalization for heart failure | 22 (37.9) | 36 (66.7) | 0.31 (0.15-0.62) | 0.001 |
| Cardiovascular death | 9 (15.5) | 19 (35.2) | 0.36 (0.15-0.86) | 0.02 |

* Hazard ratios were calculated with the use of a multivariable Cox proportional hazards regression model. CI, confidence interval; MVr, mitral valve repair.

† The rates of the composite primary outcome do not total the rates of each composite because some patients had more than one event.

Table S3. Cox regression model (multivariate analysis) for the primary outcome and secondary outcomes.

| Variables | HR | 95% CI | P-value |
| --- | --- | --- | --- |
| Primary outcome (death or hospitalization) | | | |
| LVEF <41.5% | 3.71 | 2.02-6.82 | < 0.001 |
| Atrial fibrillation | 1.87 | 1.09-3.21 | 0.02 |
| All-cause death | | | |
| LVEF <41.5% | 3.93 | 1.46-10.60 | 0.007 |
| Renal insufficiency | 4.01 | 1.63-9.86 | 0.003 |
| LVEDD >66.5 mm | 5.48 | 2.31-12.96 | < 0.001 |
| Unplanned hospitalization for heart failure | | | |
| LVEF <41.5% | 3.86 | 2.06-7.22 | < 0.001 |
| Cardiovascular death | | | |
| LVEF <41.5% | 6.02 | 1.93-18.78 | 0.002 |
| LVEDD >66.5 mm | 2.99 | 1.25-7.16 | 0.01 |

CI, confidence interval; HR, hazard ratio; LVEDD, left ventricular end-diastolic diameter; LVEF, left ventricular ejection fr
